# Whole genome resequencing revealed genomic variants and functional pathways related to adaptation in Indian yak populations

**DOI:** 10.1080/10495398.2023.2282723

**Published:** 2023-11-25

**Authors:** Amod Kumar, Mahesh Dige, Saket Kumar Niranjan, Sonika Ahlawat, Reena Arora, Aneet Kour, Ramesh Kumar Vijh

**Affiliations:** a Animal Genetics Division, ICAR-National Bureau of Animal Genetic Resources (NBAGR), Karnal, India; b Animal Genetic Resources Division, ICAR-National Bureau of Animal Genetic Resources (NBAGR), Karnal, India; c Animal Biotechnology Division, ICAR-National Bureau of Animal Genetic Resources (NBAGR), Karnal, India; d ICAR-National Research Centre on Yak, Dirang, India

**Keywords:** Yak, whole genome sequencing, genomic variants, adaptation

## Abstract

The present study aims to identify genomic variants through a whole genome sequencing (WGS) approach and uncover biological pathways associated with adaptation and fitness in Indian yak populations. A total of 30 samples (10 from each population) were included from Arunachali, Himachali and Ladakhi yak populations. WGS analysis revealed a total of 32171644, 27260825, and 32632460 SNPs and 4865254, 4429941, and 4847513 Indels in the Arunachali, Himachali, and Ladakhi yaks, respectively. Genes such as *RYR2*, *SYNE2*, *BOLA*, *HF1*, and the novel transcript *ENSBGRG00000011079* were found to have the maximum number of high impact variants in all three yak populations, and might play a major role in local adaptation. Functional enrichment analysis of genes harboring high impact SNPs revealed overrepresented pathways related to response to stress, immune system regulation, and high-altitude adaptation. This study provides comprehensive information about genomic variants and their annotation in Indian yak populations, thus would serve as a data resource for researchers working on the yaks. Furthermore, it could be well exploited for better yak conservation strategies by estimating population genetics parameters *viz*., effective population size, inbreeding, and observed and expected heterozygosity.

## Introduction

The yaks (*Poephagus grunniens*) are ruminants that are distributed in two eco-environments, *viz.*, the northwestern part of India (Himachal Pradesh and Union Territory of Ladakh) with arid dry climate and the eastern part (Arunachal Pradesh and Sikkim) with wet cold environment. Local farmers employ these animals for multiple purposes, including milk, meat, wool, fuel, and these also serve as pack animals for high-altitude transportation. Yaks can graze at an extremely high altitude and withstand extremely cold temperatures, down to −40 °C.^1^ Yaks have certain special characteristics that enable them to thrive in high-altitude environments, such as heightened environmental sensing, relatively large size of lungs, and heart.^2^

According to Indian Livestock Census (2019), the total Indian yak population is estimated to be around 58 thousands and has shown a significant decrease of 24.08 percent from the previous Livestock Census (2012).^3^ Indian yaks are divided into four groups based on their area of inhabitation; Arunachali yaks, Himachali yaks, Ladakhi yaks, and Sikkimi yaks. However, only one yak breed, that is, the Arunachali yak is registered till to date which necessitates the characterization and conservation of other yak populations. It is widely acknowledged that comprehensive molecular data on within-breed and between-breed diversity is required for successful animal genetic resource management.^4^ Additionally, deciphering the genomic variation would also serve as a valuable resource for studying complex traits in Indian yak populations.

Single nucleotide polymorphisms (SNPs) are the most common type of genetic markers^5^ and are preferred over microsatellite markers for various genomic studies. SNP maps are denser and provide more detailed information than microsatellite maps. Recent technological developments and reductions in the cost of next-generation sequencing technology provide rapid and accurate identification of SNP markers for a large number of individuals. Sivalingam et al.^6^ used a double digestion restriction-associated DNA (ddRAD) sequencing technique to find SNPs in Indian yak populations. Although ddRAD sequencing is less expensive than whole genome sequencing, it has the limitation of smaller genome analysis. Presently, whole genome sequencing (WGS) is increasingly used to uncover genetic variation,^7^ and entails deciphering the order of bases in an individual’s entire genome, aided by automated DNA sequencing procedures and computer systems to put together the massive amounts of sequence data. Additionally, WGS has an edge over SNP chips in terms of providing details on rare variants. Various researchers have used the WGS technique in yak populations around the world to explore domestication signatures,^8^ understand adaptation to high altitude,^9^ evolution and divergence,^10^ and in the development of *de novo* assembly.^11^ Moreover, the role of whole genome resequencing methods in conservation biology has also been summarized.^12^

To the best of our knowledge, this is the first study reporting genomic variant identification, annotation, and molecular pathways associated with local adaptation and fitness in Indian yak populations (Arunachali, Himachali, and Ladakhi yak) using whole genome sequencing. This study would not only provide genomic data resources for researchers working on the yaks but also help to steer future research into genetic mechanisms underlying various traits in Indian yak populations.

## Materials and methods

### Sample collection

A total of 30 yaks belonging to three different populations (10 samples from each population, that is, Himachali, Ladakhi, and Arunachali) were used for this study. The genomic DNA (gDNA) of these yaks were obtained from the DNA repository of various labs at ICAR-National Bureau of Animal Genetic Resources, Karnal, India. The quality and quantity of isolated gDNA were checked using Qubit ds DNA estimation and agarose gel electrophoresis.

### Whole genome sequencing shotgun library preparation

DNASeq libraries for all samples were prepared using the NEBNext UltraII DNA library preparation kit for Illumina; Cat No. E7770 (New England Biolabs), according to manufacturer’s recommended protocol, and sequencing was done on the S4 flowcell of the NOVASEQ 6000 using 150 bp paired-end chemistry. A commercial service provider having adequate experience in NGS data generation was selected for the library preparation and sequencing of all the DNA samples.

Briefly, 1 µg of gDNA was taken for downstream processing, and it was end-repaired and A-tailed after fragmentation followed by ligation of indexed adapters. To make the final gDNA library, the products were purified and enriched using PCR. A Qubit Fluorometer (Invitrogen, Life Technologies, Grand Island, NY, USA) was used to quantify the library. Library fragment distribution was checked on the HSDNA kit using Tapestation (Agilent Technologies, USA). The tagged gDNA libraries were mixed in equal proportions and put into the c-bot automated system for cluster generation. Post cluster generation, the libraries were put into the Illumina S4 Flow Cell of Illumina Novaseq 6000 sequencing system and 2 × 150 bp paired end chemistry was used for sequencing. Further, the sequenced samples were demultiplexed and the CASAVA v1.8.2 (Illumina Inc.) programme was used to trim the indexed adapter sequences. FastQC, a quality control tool for high throughput sequence data was then used to assess the quality of raw sequencing data.

### Read alignment, variant calling, and filtering

The Standard Broad Genomics Platform was followed for further analysis to call SNPs and indels. First, indexing of the reference genome (GCA_005887515.2) was done by BWA^13^ followed by thirty paired-end Illumina yak DNA sequenced fastq files into a unaligned BAM (Binary Alignment Map) files and marking of Illumina adapters using Picard tools. Further, SamToFastq was used to convert uBAM into fastq file and discount adapter sequences. The generated fastq files were aligned with the yak reference genome (GCA_005887515.2) using BWA-MEM to generate Sequence Alignment Map (SAM) files. MarkDuplicates from Picard was used to tag duplicate reads and output files were used to create the index “.bai” file. HaplotypeCaller of GATK version 4.2 was used to call germline SNPs and InDels with the -ERC GVCF, -G Standard and -G AS_Standard parameters. Further, GATK’s CombineGVCFs was used to combine GVCFs files of each population, followed by GenotypeGVCFs of GATK to perform joint genotyping on each group of samples pre-called with HaplotypeCaller. Hard-filtering was done with specific thresholds to remove variants that had annotation value lesser or greater than set threshold values using the -VariantFiltration command. QualByDepth (QD < 2.0), FisherStrand (FS > 60.0), StrandOddsRatio (SOR > 3.0), RMSMappingQuality (MQ < 40.0), MappingQualityRankSumTest (MQRankSum < −12.5), and ReadPosRankSumTest (ReadPosRankSum < −8.0) were used for variant filtration. Finally, generated .vcf file were used for further variant annotation study.

### Variant annotation using SnpEff

Filtered SNPs and Indels were annotated functionally by SnpEff version 5.0e.^14^ SnpEff is a toolbox that aids in the annotation of genetic variants and the prediction of functional effects. The SnpEff database LU_Bosgru_v3.0.99 was downloaded for variant annotation. SNPs were categorized based on number of effects by impact (high, low, moderate, and modifier); functional classes (missense, nonsense, and silence); type and region (downstream, exon, intergenic, intron, splice site acceptor, splice site donor, splice site region, transcript, upstream, UTR_3_prime, and UTR_5_prime).

### Gene ontology analysis

Gene ontology (GO) analysis has been a commonly used approach for functional studies of large scale genomic or transcriptomic data. GO analysis has been widely used in functional analysis and allows the identification of important categories associated with functions of interests. GO terms can provide useful information to elucidate the regulatory mechanism. Database for Annotation, Visualization and Integrated Discovery (DAVID) v6.8^15^ was used to identify over-represented biological processes for genes with high impact variants. *P*-value or EASE score along with fold enrichment parameter statistical method have been used by DAVID v6.8. which could rank the enriched terms in a more comprehensive way.

## Results

### Whole genome sequencing and read alignment

Whole genome of 30 yaks from three different native populations- Arunachali, Himachali and Ladakhi were sequenced on an average ∼10× coverage. These three different yak populations belong to different agroecological climates of India. An average of 19.9 billion reads were generated for each sample. Alignment of the reads with the yak reference genome using the Burrows-Wheeler Aligner^13^ resulted into 97.6% mapping rate and 9.5 read depth on average (Table 1).

**Table 1.** Summary of whole genome sequencing data.

Sr. No.	Population	Sample ID	Total number of sequences (R1 + R2)	Mapped percentage	Average read depth
1	Arunachal yak	1	199411832	98.77	9.66
2	Arunachal yak	2	199071166	98.83	9.54
3	Arunachal yak	3	199664536	99.32	8.95
4	Arunachal yak	4	198515614	99.02	9.25
5	Arunachal yak	5	198884010	98.75	9.62
6	Arunachal yak	6	199933222	98.77	9.78
7	Arunachal yak	7	198716592	98.80	9.594
8	Arunachal yak	9	198788874	98.59	9.57
9	Arunachal yak	10	199793478	98.83	9.52
10	Arunachal yak	16	198139854	98.84	9.58
11	Himachali yak	YAK1	198955078	98.94	9.46
12	Himachali yak	YAK2	199644652	94.31	9.460
13	Himachali yak	YAK3	198087116	99.32	8.94
14	Himachali yak	YAK4	198579582	98.67	9.81
15	Himachali yak	YAK5	198669812	98.92	9.52
16	Himachali yak	YAK6	198970780	94.41	9.44
17	Himachali yak	YAK7	199730404	94.38	9.47
18	Himachali yak	YAK8	198214482	99.03	9.39
19	Himachali yak	YAK9	198336584	94.04	9.44
20	Himachali yak	YAK10	197922834	98.99	9.41
21	Ladakhi yak	1L	198082048	98.91	9.42
22	Ladakhi yak	3L	198406104	93.69	9.74
23	Ladakhi yak	4L	199249608	93.33	9.62
24	Ladakhi yak	5L	198499154	98.85	9.36
25	Ladakhi yak	6L	198422524	93.94	9.45
26	Ladakhi yak	7L	198423912	98.78	9.49
27	Ladakhi yak	8L	198784058	98.89	9.51
28	Ladakhi yak	9L	198153712	99.26	9.02
29	Ladakhi yak	10L	198269176	99.01	9.48
30	Ladakhi yak	14L	198061058	94.23	9.33

### Variant identification

In the present investigation, a total of 32171644, 27260825, and 32632460 SNPs and, 4865254, 4429941, and 4847513 Indels were identified in the Arunachali, Himachali, and Ladakhi yak, respectively. The highest number of SNPs were found on chromosome 1, while the lowest were observed on chromosome Y. The highest number of InDels were found on chromosome 12, while the lowest number were found on chromosome Y. The chromosome-wise SNPs and InDels of these three populations have been summarized in Table 2. The differences between the number of SNPs and InDels, among the populations, provide us with raw idea related to population diversity.

**Table 2.** Chromosome-wise distribution of SNPs and InDels across the genome in Arunachali, Himachali, and Ladakhi yaks.

	Arunachali Yak		Himachali Yak		Ladakhi Yak	
Chromosome	SNPs	INDELS	SNPs	INDELS	SNPs	INDELS
1	19,52,593	2,67,216	16,13,078	2,37,296	20,01,699	2,69,813
2	16,50,035	2,25,730	13,96,542	2,03,817	17,03,494	2,29,435
3	14,89,204	2,02,837	12,45,464	1,80,901	14,97,823	2,00,571
4	14,89,233	2,07,917	12,31,159	1,83,910	14,64,339	2,01,798
5	14,85,847	2,07,948	12,88,492	1,92,029	15,45,220	2,12,870
6	14,84,394	2,10,241	12,72,334	1,91,537	15,21,977	2,12,485
7	14,08,177	1,96,112	11,60,249	1,72,089	14,12,237	1,93,253
8	13,62,983	1,91,313	11,34,844	1,70,023	13,61,662	1,88,076
9	12,80,850	1,77,682	10,79,979	1,60,376	13,07,719	1,78,169
10	12,86,280	1,82,749	10,90,354	1,65,094	13,18,726	1,84,130
11	10,93,826	1,50,296	9,29,165	1,36,716	11,08,429	1,50,202
12	9,24,736	3,13,697	7,90,042	3,00,423	9,48,823	3,14,032
13	11,15,808	1,59,886	10,04,916	1,51,724	11,48,110	1,62,138
14	9,87,500	1,38,300	8,43,491	1,25,237	10,34,177	1,42,358
15	10,98,755	1,57,070	9,40,061	1,42,676	11,32,832	1,60,987
16	9,81,463	1,40,320	8,10,222	1,23,955	9,57,817	1,35,237
17	8,57,715	1,19,096	7,31,469	1,08,197	8,96,175	1,21,297
18	8,36,835	1,15,183	7,13,528	1,04,512	8,49,911	1,14,740
19	7,75,230	1,08,692	6,63,688	99,211	7,73,160	1,05,665
20	8,10,843	1,14,949	6,91,797	1,04,141	8,18,538	1,13,358
21	7,47,722	1,02,451	6,25,787	91,553	7,85,519	1,05,158
22	7,29,550	1,02,477	6,17,403	92,627	7,57,769	1,03,234
23	6,84,525	96,030	5,82,406	87,480	6,93,612	96,207
24	8,56,003	1,24,646	7,20,168	1,11,166	7,98,056	1,15,860
25	6,27,652	87,733	5,32,079	79,482	6,33,787	86,848
26	5,66,837	80,149	4,84,970	72,794	5,64,886	78,550
27	5,69,517	81,814	4,85,716	74,063	5,75,897	81,623
28	5,21,672	71,913	4,37,820	64,160	5,35,318	72,414
29	5,81,916	2,19,462	4,95,961	2,10,713	5,61,056	2,15,506
X	13,71,166	2,30,508	11,58,401	2,16,940	13,73,658	2,20,745
Y	3,01,308	49,116	2,65,877	45,736	3,11,874	50,364

### Variant annotation

**Figure 1.**
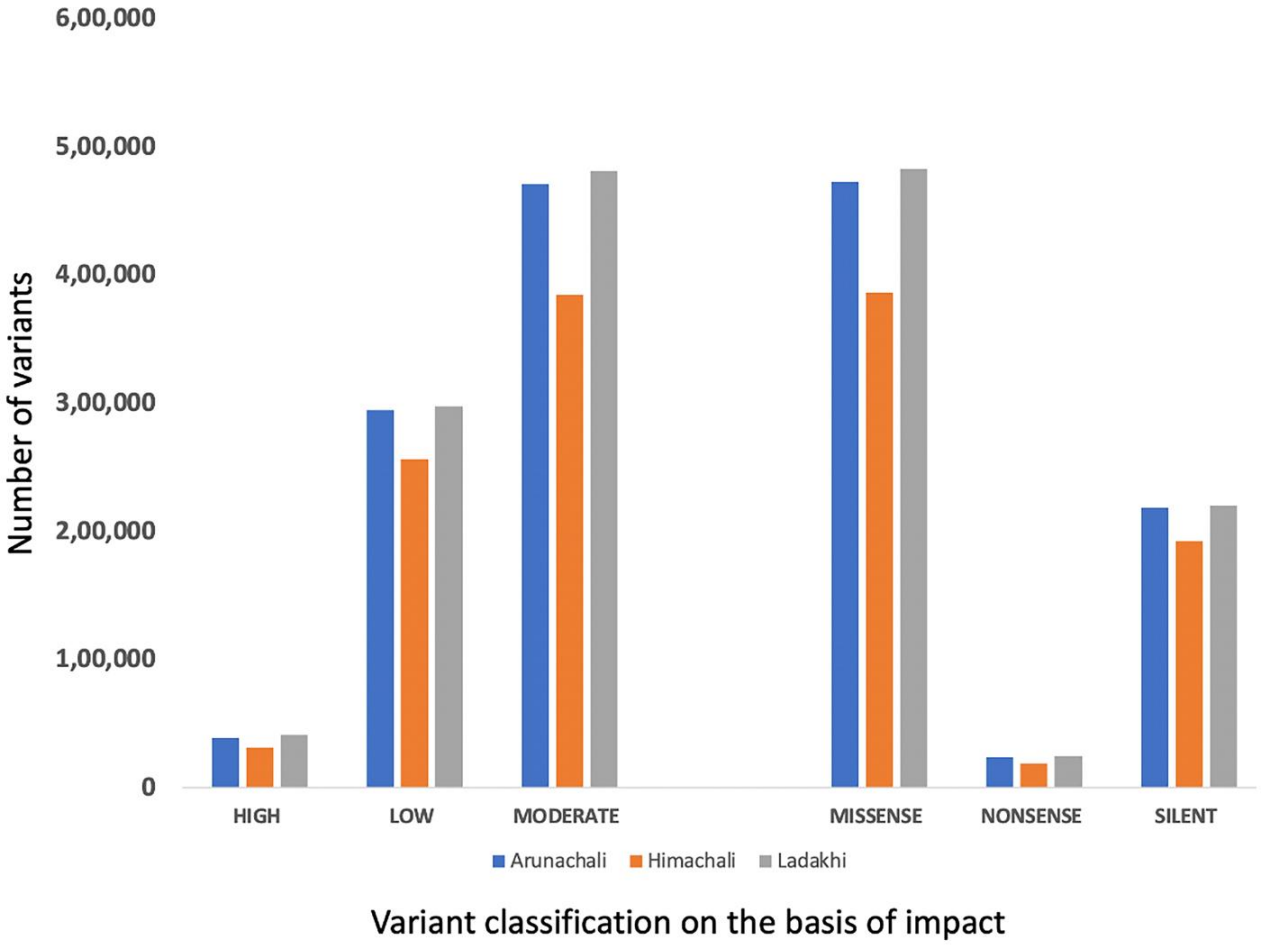
Categorization of identified SNPs in Arunachali, Himachali and Ladakhi yaks (SNPs were classified: number of effects by impact (high, low, and moderate); number of effects by functional classes (Missense, Nonsense and Silence)).

The filtered variants (SNPs and InDels) were further annotated using SnpEff.^14^ Annotation of SNPs and InDels provides detailed information regarding their position in the genome which includes intronic, untranslated region, upstream, downstream, splice site, or intergenic regions. Coding effects such as synonymous or non-synonymous amino acid replacement, start codon gains or losses, stop codon gains or losses, or frame shifts can also be predicted. The results revealed that a maximum number of SNPs and InDels were located in the intronic region in all three yak populations followed by the intergenic region. Region-specific distribution of SNPs and InDels have been mentioned in Table 3. After categorizing SNPs based on their impact, the majority fell into the modifier category (98.6%) followed by moderate, low and high (Fig. 1). However, based on the functional classes, the maximum SNPs were included under missense, followed by silent and nonsense. It is worth mentioning that *RYR2*, *SYNE2*, *BOLA*, and *HF1* genes and the novel transcript *ENSBGRG00000011079* were found to have a maximum number of high impact variants (>10) in all three yak populations.

**Table 3.** Annotation of SNPs and InDels detected in Arunachali, Himachali, and Ladakhi yaks.

Sr. No.	Region	Numbers of SNPs			Numbers of InDels		
		Arunachali	Himachali	Ladakhi	Arunachali	Himachali	Ladakhi
1.	DOWNSTREAM	2698468	2290849	2742524	436415	398208	432463
2.	EXON	791487	663930	805543	70740	63170	69640
3.	INTERGENIC	20137928	17121126	20410022	3067535	2797270	3060712
4.	INTRON	30607904	25732689	31057780	4408175	3984260	4379726
5.	SPLICE_SITE_ACCEPTOR	6591	5289	6870	3734	3570	3677
6.	SPLICE_SITE_DONOR	6455	5248	7200	1309	1217	1329
7.	SPLICE_SITE_REGION	61414	51155	63063	11832	11094	11655
8.	TRANSCRIPT	1536	1272	1528	276	249	266
9.	UPSTREAM	2671166	2268758	2706422	424340	388999	421722
10.	UTR_3_PRIME	139763	118321	141953	22339	20461	22049
11.	UTR_5_PRIME	67561	57951	68859	9244	8722	9292

### Gene ontology analysis

**Figure 2.**
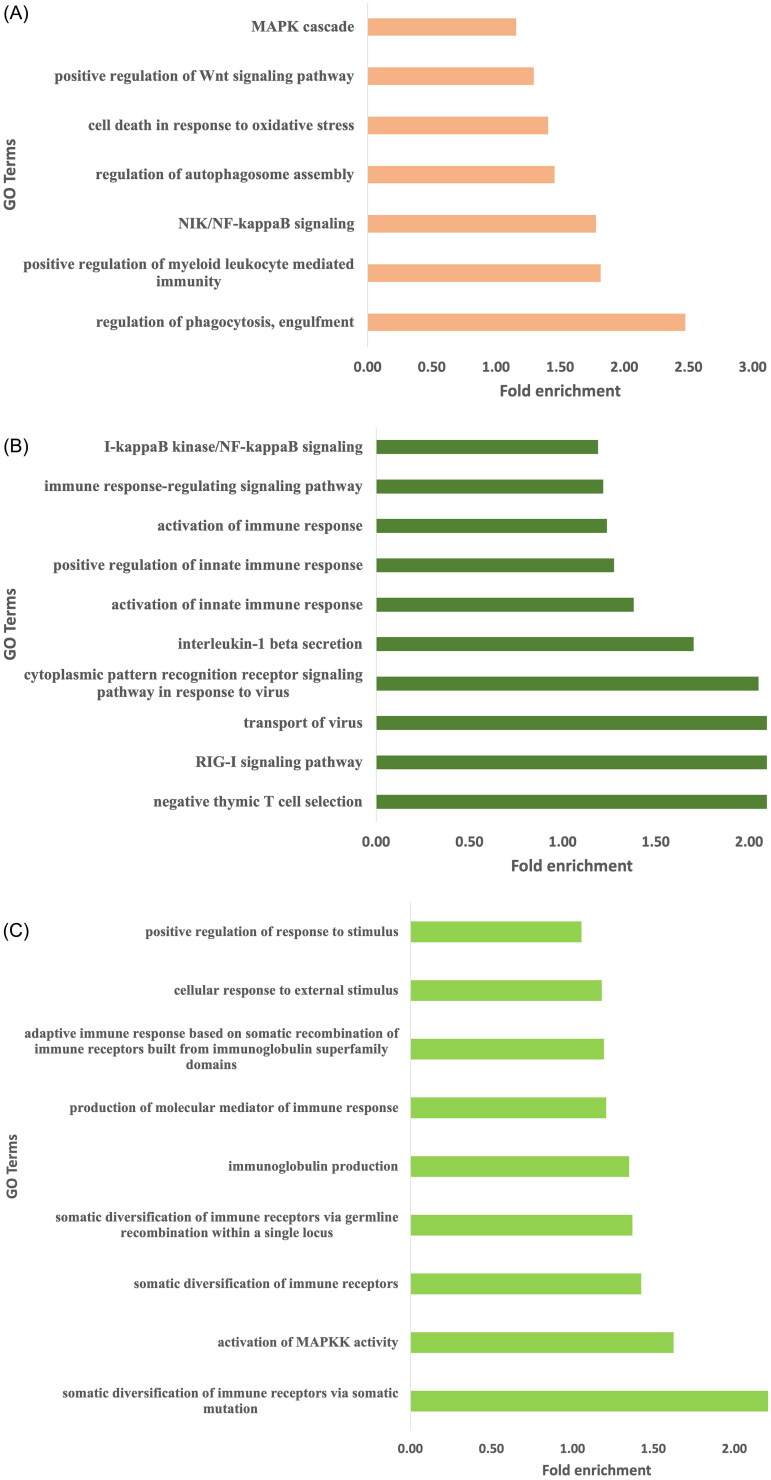
The immune related enriched GO terms observed for genes harboring high impact variants. (A) Arunachali yaks. (B) Himachali yaks. (C) Ladakhi yaks.

**Figure 3.**
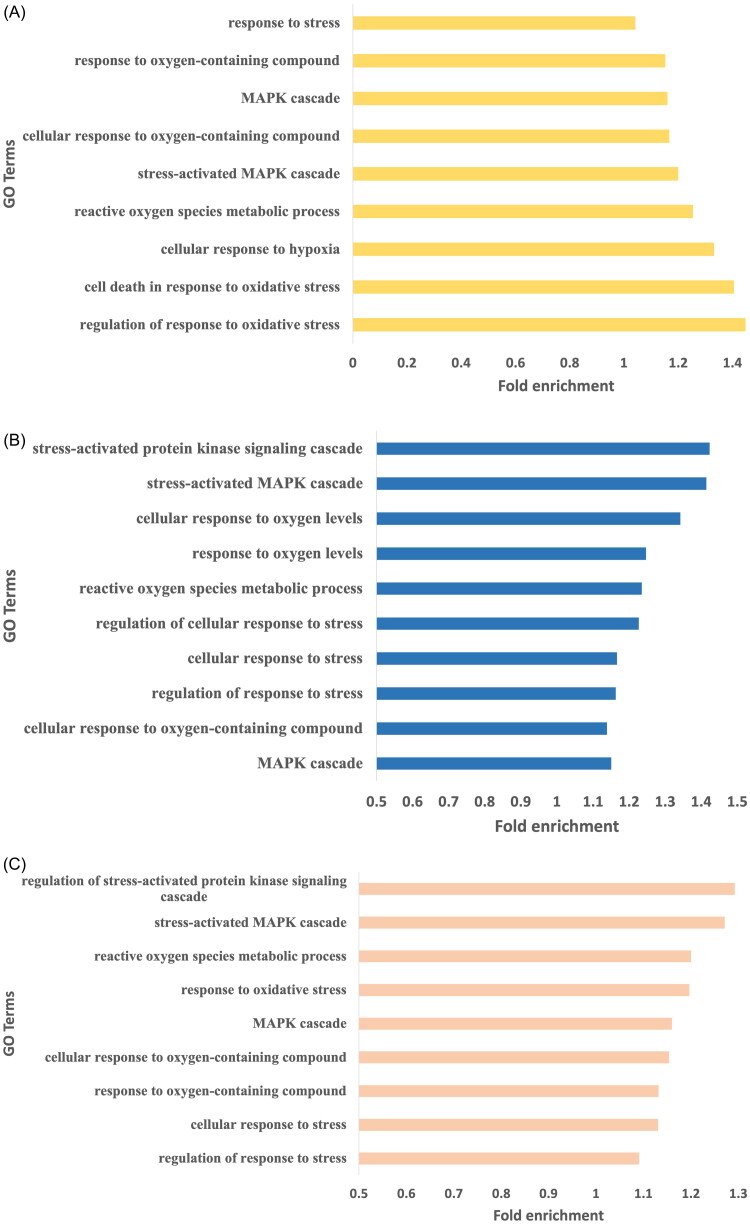
The hypoxia and stress related enriched GO terms observed for genes harboring high impact variants. (A) Arunachali yaks. (B) Himachali yaks. (C) Ladakhi yaks.

Genes harboring high impact SNPs were selected for gene ontology analysis. A total of 948, 862, and 761 biological pathways were identified in Arunachali, Himachali, and Ladakhi yaks, respectively (Supplementary Table 1; *P* < 0.05). All the biological pathways were further screened for the identification of immune-related pathways for a better understanding of fitness under local environmental conditions. Out of these three populations, the highest number of immune-related GO terms were found in Himachali yak as compared to Ladakhi and Arunachali yak populations (P < 0.05), including activation of immune response, positive regulation of innate immune response, activation of innate immune response, RIG-I signaling pathways, transport of virus, interleukin-1 beta secretion, and so on. Genes *NOD2*, *SYK*, *C3*, *STAP1*, *CARD10*, *EIF2AK2*, *RC3H2*, *HDAC7*, *ADGRG3*, *RASSF2*, *AGO1*, *RPS3*, *CHI3L1*, and *SASH1* were found to be common in all three populations. Further, the mitogen-activated protein kinase (MAPK) cascade-related pathways were significant and highly enriched in all three yak populations. Each living body cell has the basic ability to cope up and respond to changing environmental conditions. The MAPK module, a three-tiered cascade of protein kinases, is a frequently used molecular tool for inducing such responses.^16^ The identified immune-related pathways in all three populations have been summarized in Fig. 2A–C. Additionally, generated data on biological pathways was also screened to identify pathways related to local adaptation and stress. Biological pathways, response to oxygen-containing compound, cellular response to oxygen-containing compound and reactive oxygen species metabolic process were found to be enriched in all three yak populations while cellular response to hypoxia was enriched in Arunachali yak and cellular response to oxygen levels was enriched in Himachali yak. The fold enrichment of GO terms for all these yak populations has been illustrated in Fig. 3A–C.

## Discussion

In this study, a whole genome sequencing approach was used for the first time to decipher genomic variant information in Indian yak populations. The highest number of SNPs was found in Ladakhi yak (32632460), while the lowest was in Himachali yak (27260825). The number of InDels was approximately equal in the Arunachali and Ladakhi yaks, but slightly higher than the Himachali yak. Chai et al.^10^ have reported approximately 44.3 million high quality SNPs in 108 yaks using whole genome sequencing analysis (average depth of 7.2×). Similarly, Qiu et al.^8^ have identified 14.56 million SNPs across the genomic data (average depth of 6.7×) of 72 yaks. Similarly, 7.7 million quality SNPs were reported in six female *Jinchuan* yaks by whole genome sequencing (average depth of 25×, then adjusted to the sequencing depth to 6.77) approach.^17^ Our findings revealed higher numbers of SNPs as compared to previous studies in terms of numbers of animals and populations, which might be due to the lesser selection intervention, a sizable base population, and diverse populations inhabiting different climatic conditions. A previous study based on ddRAD sequencing of Indian yak populations also revealed high molecular variation within all yak populations along with considerable effective population size.^6^ Whole genome resequencing approach was used in the present study which presents an edge over previous studies related to variants information covering whole genome. This whole genome resequencing data could further be utilized for the detection of selection sweeps and copy number variations in indigenous yak populations. In our study, chromosome 1 had the maximum number of SNPs while chromosome Y had the least. However, the highest numbers of InDels were found in chromosome 12 in all the three populations. The highest and lowest numbers of SNPs on these chromosomes could be correlated with the chromosome sizes. The maximum number of SNPs and InDels were found in intronic and the intergenic region and this could be corroborated well with the previous reports.^8^^,^^18^

Annotation of identified SNPs was done which involved the categorization of variants based on their relationship with genomic sequences, and the way it may affect the gene product. To achieve this, SnpEff,^14^ predicted the effects of genetic variants by categorizing the SNPs based on putative impact (high, moderate, low, and modifier) and function (missense, non-sense, and silent). Annotation study revealed that all the three yak populations had highest numbers of SNPs in the modifier and missense categories, while the least number of SNPs were detected in the high impact and nonsense categories. Mishra et al. have also reported similar results using ddRAD approach in buffaloes.^19^ The identification of variants under the high impact and missense categories might be useful for studying their impact on complex traits. Further, the *RYR2*, *SYNE2*, *BOLA*, and *HF1* genes, and novel transcript *ENSBGRG00000011079* were observed with the highest number of high impact variants in all the three yak populations. The *RYR2* gene product has been reported to regulate intracellular calcium concentration in heart,^20^ *SYNE2* maintains the structural integrity of the nucleus and *BOLA* is involved in immune responses. Previous investigations have found indications of positive selection on *RYR2* in the Tibetan wolf, chicken, and goat, probably because of its potential function in hypoxia adaption. Inadequate oxygen delivery to the tissues induces release of reactive oxygen and an increase of intracellular calcium which ultimately induce cell contraction, a crucial response to hypoxia.^21^ Further, the immune response is significantly influenced by major histocompatibility complex (MHC), while the presence of high impact variants in *BOLA* genes are thought to play a substantial role in the diversity of antigen presenting molecules in yaks. Overall, the presence of the highest number of variants in these genes might be crucial for high altitude adaptation and associated with local fitness in Indian yaks.

Further, gene ontology analysis was performed using DAVID to identify highly enriched pathways containing genes with high impact SNPs in protein coding region. The latest version, DAVID v6.8, includes a comprehensive set of functional annotation tools to understand various biological pathways behind a large number of genes. Out of total biological pathways, Himachali yak showed the highest number of immune related significant (*P* < 0.05) biological pathways followed by Ladakhi and Arunachali yak. The fold enrichment of immune related pathways varied from 1.15–2.1, 1.06–2.21, and 1.16–2.47 in Himachali, Ladakhi, and Arunachali yak, respectively. The fold enrichment ratio measures the magnitude of enrichment, and the ratio 1.5 and above are suggested to be considered as the threshold for significant results.^15^

Genes were extracted from all immune related pathways to get more comprehensive information. Genes *NOD2*, *C3*, *CARD10*, *SYK*, *STAP1*, *EIF2AK2*, *RC3H2*, *HDAC7*, *ADGRG3*, *RASSF2*, *AGO1*, *RPS3*, *CHI3L1*, and *SASH1* were found to be common in all three yak populations. *NOD2* (Nucleotide-binding oligomerization domain 2) encodes a protein that acts as a sensor for muramyl-peptides generated from the bacterial cell wall. It is reported to be involved in innate immune regulation by activating type-I interferon and autophagy.^22^ *NOD2* was found to harbor 3, 2, and 1 high impact SNPs in Ladakhi, Himachali, and Arunachali yaks, respectively. *C3* (complement C3) plays crucial role in triggering both classical and alternative complement activation pathways and was found to contain three high impact SNPs in Ladakhi and Himachali yak while 2 SNPs in Arunachali yak. Gene, *CARD10* (caspase recruitment domain family member 10) is recognized as apoptosis signaling gene which activates NF-kappa-B through B-cell lymphoma/leukemia 10. This gene was found to harbor 1 high impact SNP in Arunachali and Himachali yak and 4 in Ladakhi yaks. However, the *RASSF2* gene involved in Ras signaling and the *AGO1gene* involved in RNA silencing were found to harbor more high impact variants in Arunachali yaks as compared to Himachali and Ladakhi yaks. *SYK* protein is widely expressed in hematopoietic cells and involved in coupling activated immunoreceptors to downstream signaling events that mediate diverse cellular responses, including proliferation, differentiation, and phagocytosis. The *SYK* gene was found to have one high impact variant in each Arunachali and Himachali yaks, while two in Ladakhi yaks. Similarly, Ladakhi yak population was found to harbor more number of high impact variants in genes *STAP-1* (2) and *HDAC7* (6) as compared to Arunachali and Himachali yaks. *STAP-1* is primarily expressed in the lymph nodes and spleen and regulates invariant natural killer T (iNKT) cells maintenance and activation,^23^ and *HDAC7* is highly expressed in CD4+/CD8+ thymocytes and functions as a signal-dependent repressor of gene transcription during T-cell development.^24^

Arunachali yak population was found to harbor more number of high impact variants in genes such as *CHI3L1* (2), *RPS3* (3), and *ADGRG3* (2) as compared to Himachali and Ladakhi yaks populations. *CHI3L1* is a chitinase-like protein that is produced in innate immune cells and extensively expressed in a variety of inflammatory disorders with infectious and noninfectious aetiologies.^25^ Similarly, *RPS3* is a multifunctional protein that also plays central role in regulating numerous aspects of host–pathogen interactions.^26^ Furthermore, Himachali yak population was found to harbor more number of high impact variants in *RC3H2* (3) gene whereas, Arunachali and Himachali yaks were found to have one in each population. *RC3H2* also defined as RING Finger And CCCH-Type Zinc Finger Domain-Containing Protein 2 is a protein coding gene that enables nucleic acid binding activity and ubiquitin protein ligase activity. Another gene *EIF2AK2*, also defined as eukaryotic translation initiation factor 2-alpha kinase 2 was found to have one high impact variant in each yak population. It is a serine/threonine-protein kinase that is IFN-induced and dsRNA-dependent and is essential for the innate immune response to viral infection.^27^

More number of significant immune-related enriched biological pathways in Arunachali and Ladakhi yaks could suggest greater genetic diversity in the set of genes behind these pathways. This could be correlated with greater fitness to combat many viral infections and better innate immunity. However, this statement needs further validation by measuring a large number of diseased phenotypes and analyzing the genomic data associated with them.

Further, biological pathways were screened in all three populations to get a better understanding of local adaptation. These populations may vary in their adaptation tolerance for lesser oxygen or hypoxic conditions and evolve with specific abilities to cope with this condition. Previous studies on animals and humans have suggested that responses to hypoxic conditions are regulated by oxygen sensing and signal transduction pathways.^28^ Ladakhi yaks are found at the highest altitude, while Arunachali yaks are found at the lowest altitude among three yak populations. Biological pathways *viz*., response to oxygen-containing compound, cellular response to oxygen-containing compound, reactive oxygen species metabolic process were found to be enriched in all three populations. However, GO terms including cellular response to hypoxia and cellular response to oxygen levels were found to be exclusively enriched in Arunachali yaks and Himachali yaks, respectively. Genes, *HIF1A* (Hypoxia-inducible factor-1alpha) and *HIF3A* were found to harbor one high impact polymorphism in protein coding region in Himachali and Arunachali yaks but were not detected in Ladakhi yaks. *HIF-1α* plays a key role in oxygen homeostasis, cellular responses to hypoxia and its abnormal expression has been reported in hypoxia related diseases.^29^ *HIF1A* helps to maintain oxygen homeostasis by stimulating glycolysis, erythropoiesis, and angiogenesis.^30^ Further, the *PDK1* (Pyruvate Dehydrogenase Kinase 1) gene associated with hypoxia and Chronic Mountain Sickness was found to contain one high impact variant in both of their transcripts (*ENSBGRT00000021747* and *ENSBGRT00000021884*) in Arunachali and Himachali yaks, while no high impact variant was found in Ladakhi yaks. The *HIF-1* transcription factor directs the expression of genes involved in adaptation to low oxygen environments. It stimulates glycolysis and represses oxygen consumption by inducing *PDK1*, which results in increased availability of oxygen and reduced cell death.^31^ Additionally, *HIF-1* also targets angiogenic agents, which induces increased blood vessels formation for the supply of more oxygenated blood to hypoxic tissue. Genes, *HMOX1* (Heme Oxygenase-1) and *VEGF* (Vascular endothelial growth factor) are downstream targets of *HIF-1α*, and both are reported to play pivotal role during hypoxic condition.^32^ Furthermore, Arunachali yaks harbor 1 and Himachali yaks harbor two high impact variants in each transcript (*ENSBGRT00000021970* and *ENSBGRT00000022054*) of the *HMOX1* gene while no high impact variant was found in Ladakhi yaks. The gene *VEGF* is a potent angiogenic factor^33^ that stimulates vascular endothelial cells and act as main mediator of hypoxia-induced-neovascularization^34^ and vascular permeability.^35^ The gene *EDN1* (endothelin 1) was also identified as a candidate gene for positive selection in the high-altitude populations.^36^ *EDN1* (Endothelins 1) was found to harbor three high impact variations in Arunachali yaks and one in Himachali yaks. However, no high impact variants were found in Ladakhi yaks. *EDN1* are endothelium-derived vasoconstrictor peptides involved in a range of functions, including biological function, cellular response to hypoxia. Here, the disparity of high impact variants in the genes (*HIF1A*, *HIF3A*, *PDK1*, *HMOX1*, and *EDN1*) of these yak populations could be associated with positive selection or their adaptation against hypoxic conditions during evolution.

Biological pathways related to the regulation of stress were found to be enriched in all three yak populations. These pathways include regulation of response to stress, cellular response to stress, response to stress, regulation of response to oxidative stress, and pathways related to the MAPK cascade. MAPK signaling pathways are known to elicit the cellular response to environmental stress. Recently, a transcriptomic study on high altitude cattle revealed MAPK signaling pathway as a highly impacted pathway.^37^ Some previous studies also established the correlation of this pathway related to high-altitude adaptations and hypoxic conditions.^38^^,^^39^ Therefore, enrichment of the above-mentioned pathways might be associated with high-altitude adaptations in Indian yaks.

## Conclusion

In conclusion, whole genome analysis revealed that Ladakhi yaks were found to possess higher SNPs than Himachali and Arunachali yaks. In all the populations considered for this study, highest numbers of SNPs were found in chromosome 1, while the lowest in on chromosome Y. The highest numbers of InDels were found in on chromosome 12, while the lowest numbers were found in on chromosome Y. In addition to the numbers, annotation of SNPs and InDels provided detailed information regarding their position in the genome which includes intronic, untranslated region, upstream, downstream, splice site, or intergenic regions. Further, the disparity of high impact variants in *HIF1A*, *HIF3A*, *PDK1*, *HMOX1*, and *EDN1* genes in these yak populations could be associated with positive selection or their high-altitude adaptation against hypoxic conditions during evolution. Genes *RYR2*, *SYNE2*, *BOLA*, and *HF1*, novel transcripts *ENSBGRG00000011079*, and *HF1* were observed with the highest number of high impact variants in all yak populations, underlining the significance of these genes and their enriched biological pathways in adaptation and fitness for their native environment. Moreover, the information generated could be well exploited for better yak conservation strategies by estimating population genetics parameters *viz.*, effective population size, inbreeding, and, observed and expected heterozygosity.

## Supplementary Material

Supplemental Material

## Data Availability

The raw data and .vcf file have been submitted to Sequence Read Archive (PRJNA803425) and European Variant Archive (PRJEB50815), respectively.
